# Analysis of Mortality in Intracerebral Hemorrhage Patients with Hyperacute Ischemic Stroke Treated Using Thrombolytic Therapy: A Nationwide Population-based Cohort Study in South Korea

**DOI:** 10.3390/jpm12081260

**Published:** 2022-07-30

**Authors:** Hyun-Young Choi, Yongil Cho, Wonhee Kim, Yang-Ki Minn, Gu-Hyun Kang, Yong-Soo Jang, Yoonje Lee, Jae-Guk Kim, Jihoon Kim, Youngsuk Cho, Hyungoo Shin, Shinje Moon, Chiwon Ahn, Juncheol Lee, Dong-Geum Shin, Jae-Keun Park

**Affiliations:** 1 Department of Emergency Medicine, Kangnam Sacred Heart Hospital, Hallym University College of Medicine, Seoul 07441, Korea; chy6049@naver.com (H.-Y.C.); yong0831@naver.com (Y.L.); gallion00@gmail.com (J.-G.K.); 2 Hallym Biomedical Informatics Convergence Research Center, Kangnam Sacred Heart Hospital, Hallym University College of Medicine, Seoul 07441, Korea; emkang@hallym.or.kr (G.-H.K.); amicoys@hallym.or.kr (Y.-S.J.); 3 Department of Emergency Medicine, Hanyang University College of Medicine, Seoul 04763, Korea; joeguy@hanyang.ac.kr (Y.C.); seodtst@gmail.com (H.S.); jclee0221@gmail.com (J.L.); 4 Department of Neurology, Kangnam Sacred Heart Hospital, Hallym University College of Medicine, Seoul 07441, Korea; yangki2@hallym.or.kr; 5 Department of Thoracic and Cardiovascular Surgery, Kangnam Sacred Heart Hospital, Hallym University College of Medicine, Seoul 07441, Korea; jkim@hallym.ac.kr; 6 Department of Emergency Medicine, Kangdong Sacred Heart Hospital, Hallym University College of Medicine, Seoul 05355, Korea; faith2love@hanmail.net; 7 Department of Internal Medicine, Kangnam Sacred Heart Hospital, Hallym University College of Medicine, Seoul 07441, Korea; sinjei1129@gmail.com (S.M.); blaudg@naver.com (D.-G.S.); hanyangjj@gmail.com (J.-K.P.); 8 Department of Emergency Medicine, College of Medicine, Chung-Ang University, Seoul 06973, Korea; cahn@cau.ac.kr

**Keywords:** ischemic stroke, thrombolytic therapy, cerebral hemorrhage, mortality, cohort studies

## Abstract

This study investigated the impact of intracerebral hemorrhage (ICH) on the cumulative mortality of patients with hyperacute ischemic stroke. This population-based retrospective cohort study used claims data from the National Health Insurance Service customized database of South Korea. The recruitment period was 2005–2018. The study population included patients with hyperacute ischemic stroke who had received intravenous thrombolysis. The primary endpoint was 12-month cumulative mortality, which was analyzed in both the ICH and no-ICH groups. Of the 50,550 patients included, 2567 (5.1%) and 47,983 (94.9%) belonged to the ICH and no-ICH groups, respectively. In the univariable analysis for 12-month mortality, ICH patients were substantially more prevalent among dead patients than among patients who survived (11.6% versus 3.6%; *p* < 0.001). The overall 12-month cumulative mortality rate was 18.8%. Mortality in the ICH group was higher than that in the no-ICH group (42.8% versus 17.5%; *p* < 0.001). In the multivariable analysis, the risk of 12-month cumulative mortality was 2.97 times higher in the ICH group than in the no-ICH group (95% confidence interval, 2.79–3.16). The risk of 12-month cumulative mortality in hyperacute ischemic stroke can increase approximately threefold after the occurrence of spontaneous ICH following intravenous thrombolysis.

## 1. Introduction

Ischemic stroke affects 700,000 patients in the United States each year [1,2,3]. An irreversible brain injury occurs if spontaneous blood flow is not restored immediately after an ischemic stroke, resulting in permanent neurological disability [3]. The most crucial treatment for ischemic stroke is thrombolytic therapy for prompt restoration of cerebral blood flow. Alteplase is the only recombinant tissue plasminogen activator (rtPA) that has been approved by the Food and Drug Administration (FDA) for use in thrombolytic therapy for patients with hyperacute ischemic stroke (HIS) [1,4,5]. Numerous studies have demonstrated the benefits of alteplase in improving neurological prognosis following HIS [2,6,7,8]; however, the drug has high bleeding potency and its administration increases the risk of intracerebral hemorrhage (ICH) [4,8,9,10]. 

As a result, research protocols on the association between rtPA administration and ICH in patients with HIS have been performed previously [9,11,12]. The incidence of ICH in HIS patients within 7 days of rtPA administration was reported as 3–10% [13,14]. Neurological symptoms that worsen after rtPA administration, abnormal findings on imaging tests, and the time between symptom onset and rtPA injection are all important factors in evaluating the prognosis of patients with rtPA-induced spontaneous ICH [2,8,9,11,15]. 

Previous large-scale studies relied mostly on registry data, and mortality was estimated over a 30- or 90-day period [13,16,17]. However, no study has used a nationwide cohort or measured mortality for >90 days.

We investigated the incidence and cumulative mortality of ICH in patients with HIS by using national data from the National Health Insurance Service (NHIS) in South Korea. 

## 2. Materials and Methods

### 2.1. Study Design and Setting

We conducted a population-based retrospective cohort study using claims data from the NHIS customized database in South Korea (NHIS-2021–1–268) (https://nhiss.nhis.or.kr/bd/ab/bdaba012eng.do (accessed on 29 June 2022)). The NHIS is a nationwide insurance program that covers 97% of the 50 million people in South Korea [18,19]. The NHIS database contains patient demographic data, diagnoses, drug prescriptions, and dates of death [19]. The diagnoses were based on the International Classification of Diseases, 10th Revision (ICD-10) codes. This study was approved by the Institutional Review Board of Kangnam Sacred Heart Hospital at Hallym University (Seoul, Republic of Korea) and the requirement for informed consent was waived (IRB No. 2019-11-022-007).

### 2.2. Study Population and Variables

This study included patients diagnosed with HIS between 2005 and 2018. We defined HIS patients as patients who were diagnosed with ischemic stroke (ICD-10: I63.x and I64.x) and received rtPA prescription codes during hospitalization for ischemic stroke. Subjects were divided into groups based on whether or not they developed spontaneous ICH (ICH group and no-ICH group, respectively). The ICH group was defined as having an ICH code (I60.x–I62.x) during hospitalization for ischemic stroke within 7 days of rtPA administration. 

The cumulative mortality of the ICH and no-ICH groups was the main outcome variable of interest in this study. The primary endpoint was the 12-month cumulative mortality. The secondary endpoints were the 1-, 3-, and 6-month cumulative mortality rates. The covariates included age, sex, and comorbidities. Comorbidities included hypertension, diabetes mellitus (DM), dyslipidemia, previous acute myocardial infarction (AMI), congestive heart failure (CHF), atrial fibrillation (AF), peripheral vascular disease, ischemic stroke, hemorrhagic stroke, chronic kidney disease (CKD), liver cirrhosis (LC), cancer, and chronic obstructive pulmonary disease (COPD). Comorbidities were defined as having two or more of the same diagnostic codes for three years before the index date (Supplementary Table S1). 

### 2.3. Analysis

For continuous variables, a normality test was performed using the Anderson–Darling test. Variables with normality are presented as the mean and standard deviation, and variables with a non-normal distribution are presented as the median and the 25–75th percentile. Student’s t-test or Wilcoxon rank-sum test were performed as appropriate. Categorical variables were presented as numbers and percentages. The chi-square test was performed to compare the categorical variables between the two groups. Cumulative mortality was estimated using the Kaplan–Meier method and was equal to one minus the overall survival probability at that time. The cumulative mortality of all patients with HIS was confirmed. Thereafter, the difference in cumulative mortality between the ICH and no-ICH groups was compared using the log-rank test.

Cox proportional hazard regression analysis was used to determine the effect of ICH after rtPA administration on cumulative mortality. As crude analysis, hazard ratios (HRs) and 95% confidence intervals (CIs) for 1-, 3-, 6-, and 12-month cumulative mortality in the ICH group alone were confirmed. Multivariable analysis was performed, including covariates for which cumulative mortality showed a significant difference in the univariable analysis. Adjusted HRs (aHRs) and 95% CIs were calculated by performing multivariable Cox hazard regression analysis. Statistical significance was determined using two-sided tests, with significance indicated by a *p* value of less than 0.05. All statistical analyses were conducted using SAS version 9.4 (SAS Institute Inc., Cary, NC, USA) and R version 3.5.2 (www.R-project.org (accessed on 29 June 2022)).

## 3. Results

### 3.1. Characteristics of Patients

We enrolled 50,550 patients with HIS treated with rtPA. The number of patients in the ICH and no-ICH groups was 2567 (5.1%) and 47,983 (94.9%), respectively. The baseline characteristics of the study population are summarized in Table 1. The median age was 69 years for both the groups (25–75th percentile: 60–76 and 59–75 for ICH and no-ICH groups, respectively). The proportion of male patients was significantly higher than that of female patients in both ICH (60.1%) and no-ICH (61.9%) groups. The most common comorbidities were hypertension, dyslipidemia, and DM.

**Figure 1:**
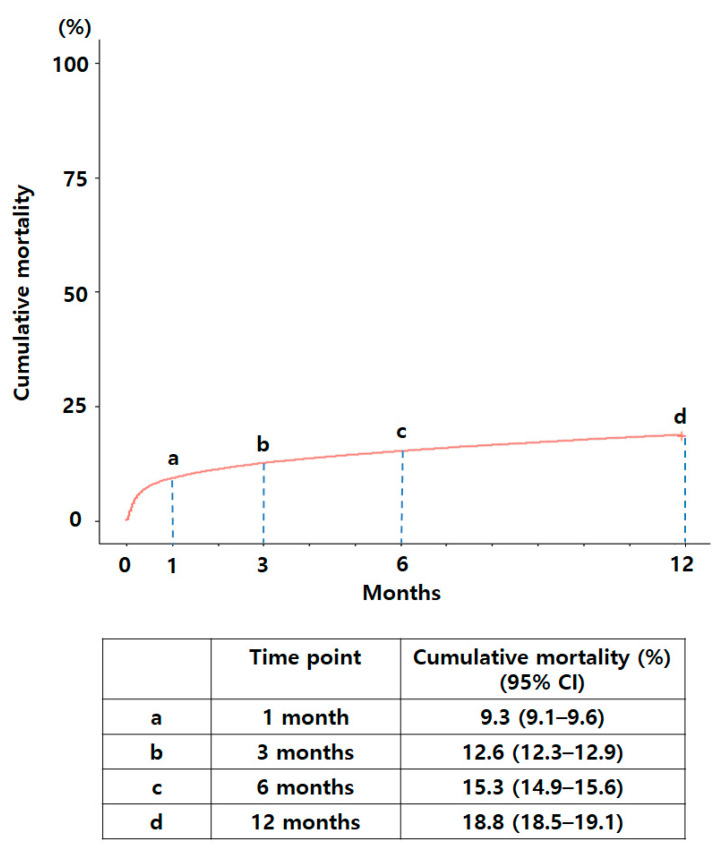
The 1-, 3-, 6-, and 12-month cumulative mortality rates of the total study population.

**Figure 2:**
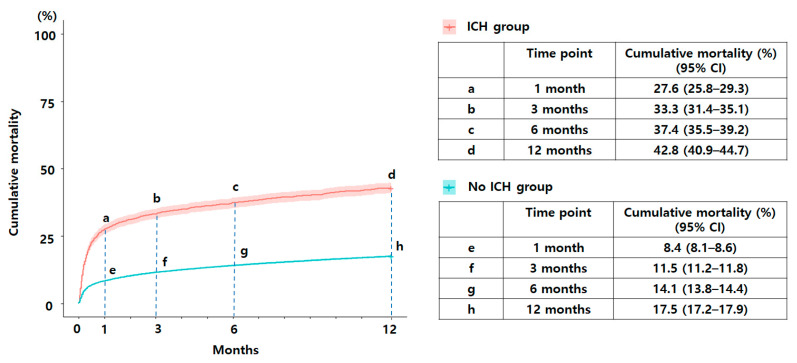
The 1-, 3-, 6-, and 12-month cumulative mortality rates according to the occurrence of spontaneous intracerebral hemorrhage.

The overall 1-, 3-, 6-, and 12-month cumulative mortality rates of the study population were 9.3%, 12.6%, 15.3%, and 18.8%, respectively (Figure 1). The 12-month cumulative mortality was higher in the ICH group than in the no-ICH group (42.8% versus 17.5%; *p* < 0.001; Figure 2). The 1-, 3-, and 6-month cumulative mortality rates of the ICH group were 27.6%, 33.3%, and 37.4%, respectively. The 1-, 3-, and 6-month cumulative mortality rates of the no-ICH group were 8.4%, 11.5%, and 14.1%, respectively.

### 3.2. Univariable Analysis for Cumulative Mortality between ICH vs. No-ICH Group

The proportion of patients with ICH in the group of patients who did not survive 12 months was significantly higher than that in the survival group (11.6% versus 3.6%; *p* < 0.001) (Table 2). Patients in the death group were older than those in the survival group (median age, 67 versus 75 years; *p* < 0.001). The proportion of women in the death group was higher than that in the survival group (46.2% versus 36.3%; *p* < 0.001). The proportion of all comorbidities was significantly higher in the death group than in the survival group. 

Similarly, for the 1-, 3-, and 6-month cumulative mortality, the proportion of ICH patients was higher in the death group than in the survival group (Supplementary Tables S2–S4). Univariable analysis of 1-, 3-, and 6-month cumulative mortality showed that patients were older and the proportions of women and comorbidities were higher in the mortality group than in the survival group. However, univariable analysis of 1-month cumulative mortality showed that the proportion of patients with comorbidities, such as hemorrhagic stroke and liver cirrhosis, did not differ significantly between the death and survival groups.

### 3.3. Multivariable Analysis for Cumulative Mortality between ICH vs. No-ICH Group

Multivariable analysis was performed to determine the extent to which the risk of death was higher in the ICH group than in the no-ICH group. After adjusting for significant factors in the univariable analysis, the risk of 12-month cumulative mortality was 2.97 times higher in the ICH-group than in the no-ICH group (95% CI, 2.79–3.16) (Table 3 and Table 4). In the ICH-group, the risk of death was higher with shorter follow-up periods than in the no-ICH group; the aHR (95% CI) for 6-, 3-, and 1-month cumulative mortality was 3.10 (2.89–3.31), 3.26 (3.03–3.50), and 3.52 (3.25–3.81), respectively.

## 4. Discussion

In this study, we found that when ICH occurred in HIS patients treated with rtPA, the 12-month cumulative mortality rate was 2.97 times higher than in patients who did not have ICH. Several previous studies on the frequency of occurrence and associated factors of spontaneous ICH after rtPA administration have been conducted, but most have been analyzed primarily using registries, and no national cohort study has been conducted on the entire population of a country over a period of more than 10 years [2,8,9,12,13]. The 12-month cumulative mortality rate was measured for the first time in this study. The mortality rate in the ICH group was approximately three times greater than in the no-ICH group after 12 months of rtPA administration.

We used the NHIS data from 2005 to 2018 to measure the incidence rate of spontaneous ICH in patients with HIS and the long-term mortality associated with the incidence of spontaneous ICH in HIS for all patients in South Korea. We utilized NHIS data for a variety of reasons. First, the date of diagnosis and ICD-10 code for diagnosis could be found. Second, the date of administration of any medicine, including rtPA, could be determined. Third, the exact date of the patient's death could be identified [18,20,21]. The NHIS database is a national database that contains data covering a long period of time, as well as the diagnosis and death dates; it is ideal for investigating long-term mortality. Furthermore, by tracking each patient's ICD-10 code, their medical history can be confirmed.

In previous studies, diverse time standards have been used to define ICH that occurred after rtPA administration [13]. In this study, we defined spontaneous ICH as ICH occurring within 7 days of rtPA administration, in line with previous studies [2,15,22]. No previous study has examined the effect of ICH occurrence on mortality in relation to other variables, including underlying disease for 12 months. 

There was no significant difference in the rate of ICH occurrence in a previous large-scale study (SITS-ISTR) in which more than 10,000 patients were administered rtPA [16,23,24]. However, the major determinants of ICH vary among the studies. Tong et al. showed that ICH occurrence significantly increases hypertension, dyslipidemia, DM, and AF [1]. Xue et al. reported that the incidence of ICH was high in patients with coronary heart disease [12], and Zhang et al. found that the incidence of ICH was low in AF and had no effect on hypertension or DM [9]. The results of the factors associated with the occurrence of ICH were not consistent across the previously studied cohort. In this study, hypertension, CHF, AF, and previous hemorrhagic stroke were important factors influencing the incidence of spontaneous ICH (Table 1). This study used national data over a lengthy period; therefore, the present results may be different from those of previous studies. 

More than 300 patients in the no-ICH group had hemorrhagic stroke, a condition that was contraindicated with rtPA treatment. We suspect that the following situations, which do not confirm ICH's prior history, can lead to the unintentional administration of rtPA: transfer to a hospital without any medical records, and patients whose mental state has changed. 

The 90-day mortality rate of HIS patients treated with rtPA ranged from 8% to 25% in previous studies [15,22,23,25,26,27,28]. In this study, the 90-day mortality rate was 12.6%, which was not substantially different from previous studies, whereas the 12-month mortality rate of HIS patients treated with rtPA was 18.8%, which has not been explored previously in other studies. As illustrated in Figure 1, half of the patients who died within a year of receiving rtPA died within the first month. This result may be attributable to the risk of severe side effects of rtPA, such as an increased tendency to bleeding [29,30,31]. 

When rtPA was administered to HIS patients, the presence of spontaneous ICH significantly influenced the 12-month mortality rate (Table 3). Numerous underlying diseases were significant in terms of the 12-month mortality rate in univariable analysis. Multivariable analysis performed after statistical correction revealed that the aHR was substantial in the spontaneous ICH group (2.97).

However, no previous studies have examined the long-term cumulative mortality rate over a year based on the presence or absence of ICH. The ICH group showed a significantly higher 12-month mortality rate than the no-ICH group (42.8% versus 17.5%; *p* < 0.001). As shown in Figure 2, the cumulative mortality rate in both groups increased substantially at 1 month and then gradually increased over time (ICH versus no-ICH, 42.8% versus 17.5%). The cumulative mortality rate for 1, 3, 6, and 12 months was compared between the ICH and no-ICH groups; the aHR was highest at 1 month, at 3.52, and the mortality rate at 12 months was 2.97 times higher in the ICH group than in the no-ICH group. Furthermore, the ICH group showed substantially higher mortality rates than the no-ICH group for all four time periods. We believe that neurological decline in the symptomatic ICH group might have initially contributed to the high mortality rate. A systematic review by Seet et al. showed that symptomatic ICH is a fatal complication of intravenous thrombolysis therapy and is related with a high mortality rate (measuring as far as 90-day mortality). A moderate correlation was observed between the incidence of symptomatic ICH and mortality in patients treated with intravenous thrombolysis [13].

This study has several limitations. First, because the data provided by NHIS only include diagnostic and prescription codes, it is impossible to discriminate between symptomatic and asymptomatic ICH, as well as ICH area and size in the brain. Consequently, the influence of ICH-related data on mortality could not be determined. Second, despite the fact that rtPA was successfully administered based on a National Institutes of Health Stroke Scale score in the range of 4–25, data indicating neurological changes after rtPA administration were lacking in the NHIS database. Third, owing to the lack of relevant data in the NHIS database, we were unable to assess the influence of rtPA administration time on mortality. In South Korea, rtPA is typically administered to patients within 3–4.5 h of the onset of neurological symptoms. We believe that rtPA was appropriately administered to the majority of patients because the government rigorously reviews and manages the appropriate administration period of rtPA. However, the effect of hourly changes in administration timing on mortality has not yet been studied.

## 5. Conclusions

The incidence of spontaneous ICH with rtPA treatment gradually and significantly contributed to the mortality of HIS patients over a year. Compared to HIS patients without ICH, those with ICH showed an approximately threefold higher risk of 12-month cumulative mortality. 

## Figures and Tables

**Table 1:** Baseline characteristics of the study population.

	ICH Group (*n* = 2567)	No-ICH Group (*n* = 47,983)	*p*-Value *
*Age, median [25–75th percentile]*	69 [60–76]	69 [59–75]	0.017
*Sex, n (%)*			0.07
Male	1543 (60.1)	29,723 (61.9)	
Female	1024 (39.9)	18,260 (38.1)	
*Comorbidities, n (%)*			
Hypertension	1704 (66.4)	29,700 (61.9)	<0.001
Diabetes mellitus	848 (33.0)	15,152 (31.6)	0.19
Dyslipidemia	1149 (44.8)	20,639 (43.0)	0.09
Acute myocardial infarction	85 (3.3)	1274 (2.7)	0.052
Congestive heart failure	617 (24.0)	9163 (19.1)	<0.001
Atrial fibrillation	540 (21.0)	7003 (14.6)	<0.001
Peripheral vascular disease	403 (15.7)	7525 (15.7)	1.00
Ischemic stroke	374 (14.6)	7283 (15.2)	0.42
Hemorrhagic stroke	0 (0)	350 (0.7)	<0.001
Chronic kidney disease	95 (3.7)	1489 (3.1)	0.10
Liver cirrhosis	20 (0.8)	457 (1.0)	0.44
Cancer	233 (9.1)	3962 (8.3)	0.15
COPD	169 (6.6)	2878 (6.0)	0.24

* The Wilcoxon rank-sum test was used for continuous variables. The chi-squared test was used for categorical variables. ICH, intracerebral hemorrhage; COPD, chronic obstructive pulmonary disease.

**Table 2:** Univariable analysis of attributing factors for 12-month mortality.

	Survival (*n* = 41,044)	Death (*n* = 9506)	*p*-Value *
*ICH group, n (%)*	1468 (3.6)	1099 (11.6)	<0.001
*Age, median [25–75th percentile]*	67 [58–74]	75 [68–79]	<0.001
*Sex, n (%)*			<0.001
Male	26,148 (63.7)	5118 (53.8)	
Female	14,896 (36.3)	4388 (46.2)	
*Comorbidities, n (%)*			
Hypertension	24,265 (59.1)	7139 (75.1)	<0.001
Diabetes mellitus	12,272 (29.9)	3728 (39.2)	<0.001
Dyslipidemia	17,380 (42.3)	4408 (46.4)	<0.001
Acute myocardial infarction	937 (2.3)	422 (4.4)	<0.001
Congestive heart failure	6866 (16.7)	2914 (30.7)	<0.001
Atrial fibrillation	5487 (13.4)	2056 (21.6)	<0.001
Peripheral vascular disease	6166 (15.0)	1762 (18.5)	<0.001
Ischemic stroke	5648 (13.8)	2009 (21.1)	<0.001
Hemorrhagic stroke	261 (0.6)	89 (0.9)	0.002
Chronic kidney disease	1009 (2.5)	575 (6.0)	<0.001
Liver cirrhosis	353 (0.9)	124 (1.3)	<0.001
Cancer	2959 (7.2)	1236 (13.0)	<0.001
COPD	2084 (5.1)	963 (10.1)	<0.001

* Wilcoxon rank-sum test was performed for continuous variables. The chi-squared test was used for categorical variables. ICH, intracerebral hemorrhage; COPD, chronic obstructive pulmonary disease.

**Table 3:** Multivariable analysis of attributing factors for 12-month mortality.

Factors *	aHR	95% CI	*p*-Value *
ICH group	2.97	2.79–3.16	<0.001
Age, years	1.06	1.05–1.06	<0.001
Female	1.12	1.08–1.17	<0.001
Hypertension	1.17	1.10–1.23	<0.001
Diabetes mellitus	1.16	1.11–1.21	<0.001
Dyslipidemia	0.76	0.73–0.80	<0.001
Acute myocardial infarction	1.38	1.25–1.52	<0.001
Congestive heart failure	1.33	1.26–1.39	<0.001
Atrial fibrillation	1.10	1.05–1.17	<0.001
Peripheral vascular disease	0.94	0.79–0.99	0.022
Ischemic stroke	1.19	1.13–1.26	<0.001
Hemorrhagic stroke	1.22	0.99–1.51	0.06
Chronic kidney disease	1.57	1.44–1.71	<0.001
Liver cirrhosis	1.20	1.001–1.43	0.048
Cancer	1.42	1.34–1.51	<0.001
COPD	1.25	1.17–1.34	<0.001

* Multivariable analysis was applied to statistically significant factors in the univariable analysis. * Cox proportional hazard regression was performed in the multivariable analysis for all included factors. ICH, intracerebral hemorrhage; COPD, chronic obstructive pulmonary disease; aHR, adjusted hazard ratio; CI, confidence interval.

**Table 4:** Analysis of cumulative mortality for intracerebral hemorrhage patients.

	Crude Model			Multivariable Model		
Months	HR	95% CI	*p*-Value *	aHR	95% CI	*p*-Value *
1	3.65	3.37–3.95	<0.001	3.52	3.25–3.81	<0.001 ^†^
3	3.33	3.10–3.58	<0.001	3.26	3.03–3.50	<0.001 ^‡^
6	3.14	2.94–3.36	<0.001	3.10	2.89–3.31	<0.001 ^‡^
12	2.98	2.80–3.17	<0.001	2.97	2.79–3.16	<0.001 ^‡^

* Cox proportional hazard regression analysis was performed for multivariable analysis. ^†^ Adjusted for age, sex, hypertension, diabetes mellitus, dyslipidemia, acute myocardial infarction, congestive heart failure, atrial fibrillation, peripheral vascular disease, ischemic stroke, chronic kidney disease, cancer, and chronic obstructive pulmonary disease. ^‡^ Adjusted for age, sex, hypertension, diabetes mellitus, dyslipidemia, acute myocardial infarction, congestive heart failure, atrial fibrillation, peripheral vascular disease, ischemic stroke, hemorrhagic stroke, chronic kidney disease, liver cirrhosis, cancer, and chronic obstructive pulmonary disease. HR, hazard ratio; CI, confidence interval; aHR, adjusted hazard ratio.

## Data Availability

Not applicable.

## Supplementary Materials

The following supporting information can be downloaded at: https://www.mdpi.com/article/10.3390/jpm12081260/s1, Table S1: Definitions of comorbidities defined using ICD-10 codes; Table S2: Univariable analysis of attributing factors for 1-month mortality; Table S3: Univariable analysis of attributing factors for 3-month mortality; Table S4: Univariable analysis of attributing factors for 6-month mortality.
