# Supplementary material for: Analysis of Mortality in Intracerebral Hemorrhage Patients with Hyperacute Ischemic Stroke Treated Using Thrombolytic Therapy: A Nationwide Population-based Cohort Study in South Korea
Source: J Pers Med. 2022 Jul 30;12(8):1260. doi: 10.3390/jpm12081260 (PMC9410217; doi:10.3390/jpm12081260)
Supplement: Supplementary file 1 [file jpm-12-01260-s001.zip › jpm-1817095-supplementary.pdf]

Supplementary Table S1. Definitions of comorbidities defined using ICD-10 codes

Comorbidity	ICD-10 code
Hypertension	I10-13, I15
Diabetes mellitus	E10-E14
Dyslipidemia	E78
Acute myocardial infarction	I21, I22
Congestive heart failure	I11, I13.0, I13.2, I50, J81
Atrial fibrillilation	I48
Peripheral vascular disease	I73, I77.1
Ischemic stroke	I63, I64
Hemorrhagic stroke	I60-I62
Chronic kidney disease	I12.0, I13.1, N18, N19, Z49, Z94.0, Z99.2
Liver cirrhosis	K70.2, K70.3, K74
Cancer	C00-C99
COPD	J44

ICD-10=International Classification of Diseases, 10th Revision; COPD=chronic obstructive pulmonary disease.

* Comorbidities were defined as having two or more of the same diagnostic codes for three years before the index date.

Supplementary Table S2. Univariable analysis of attributing factors for 1-month mortality

	Survival (n=41,044)	Death (n=9,506)	P-value*
ICH group, n (%)	1,865 (4.1)	702 (15.0)	<0.001
Age, median [25–75th percentile]	68 [59-75]	73 [65-78]	<0.001
Sex, n (%)			<0.001
Male	28,878 (63.0)	2,388 (51.0)	
Female	16,990 (37.0)	2,294 (49.0)	
Comorbidities, n (%)			
Hypertension	27,928 (60.9)	3,476 (74.2)	<0.001
Diabetes mellitus	14,216 (31.0)	1,784 (38.1)	<0.001
Dyslipidemia	19,589 (42.7)	2,199 (47.0)	<0.001
Acute myocardial infarction	1,151 (2.5)	208 (4.4)	<0.001
Congestive heart failure	8,375 (18.3)	1,405 (30.0)	<0.001
Atrial fibrillation	6,517 (14.2)	1,026 (21.9)	<0.001
Peripheral vascular disease	7,080 (15.4)	848 (18.1)	<0.001
Ischemic stroke	6,771 (14.8)	886 (18.9)	<0.001
Hemorrhagic stroke	309 (0.7)	41 (0.9)	0.135
Chronic kidney disease	1,316 (2.9)	268 (5.7)	<0.001
Liver cirrhosis	424 (0.9)	53 (1.1)	0.187
Cancer	3,702 (8.1)	493 (10.5)	<0.001
COPD	2,663 (5.8)	384 (8.2)	<0.001

*Wilcoxon rank-sum test was performed for continuous variables. The chi-squared test was used for categorical variables. ICH, intracerebral hemorrhage; COPD, chronic obstructive pulmonary disease

Supplementary Table S3. Univariable analysis of attributing factors for 3-month mortality

	Survival (n=41,044)	Death (n=9,506)	P-value*
ICH group, n (%)	1,714 (3.9)	853 (13.4)	<0.001
Age, median [25–75th percentile]	68 [58-75]	74 [66-79]	<0.001
Sex, n (%)			<0.001
Male	27,905 (63.2)	3,361 (52.8)	
Female	16,274 (36.8)	3,010 (47.2)	
Comorbidities, n (%)			
Hypertension	26,656 (60.3)	4,748 (74.5)	<0.001
Diabetes mellitus	13,546 (30.7)	2,454 (38.5)	<0.001
Dyslipidemia	18,853 (42.7)	2,935 (46.1)	<0.001
Acute myocardial infarction	1,079 (2.4)	280 (4.4)	<0.001
Congestive heart failure	7,867 (17.8)	1,913 (30.0)	<0.001
Atrial fibrillation	6,194 (14.0)	1,349 (21.2)	<0.001
Peripheral vascular disease	6,782 (15.4)	1,146 (18.0)	<0.001
Ischemic stroke	6,389 (14.5)	1,268 (19.9)	<0.001
Hemorrhagic stroke	286 (0.6)	64 (1.0)	0.002
Chronic kidney disease	1,207 (2.7)	377 (5.9)	<0.001
Liver cirrhosis	394 (0.9)	83 (1.3)	<0.001
Cancer	3,406 (7.7)	789 (12.4)	<0.001
COPD	2,442 (5.5)	605 (9.5)	<0.001

*Wilcoxon rank-sum test was performed for continuous variables. The chi-squared test was used for categorical variables. ICH, intracerebral hemorrhage; COPD, chronic obstructive pulmonary disease

Supplementary Table S4. Univariable analysis of attributing factors for 6-month mortality

	Survival (n=41,044)	Death (n=9,506)	P-value*
ICH group, n (%)	1,608 (3.8)	959 (12.4)	<0.001
Age, median [25–75th percentile]	67 [58-75]	74 [67-79]	<0.001
Sex, n (%)			<0.001
Male	27,178 (63.4)	4,088 (53.1)	
Female	15,668 (36.6)	3,616 (46.9)	
Comorbidities, n (%)			
Hypertension	25,637 (59.8)	5,767 (74.9)	<0.001
Diabetes mellitus	13,013 (30.4)	2,987 (38.8)	<0.001
Dyslipidemia	18,229 (42.5)	3,559 (46.2)	<0.001
Acute myocardial infarction	1,017 (2.4)	342 (4.4)	<0.001
Congestive heart failure	7,436 (17.4)	2,344 (30.4)	<0.001
Atrial fibrillation	5,884 (13.7)	1,659 (21.5)	<0.001
Peripheral vascular disease	6,508 (15.2)	1,420 (18.4)	<0.001
Ischemic stroke	6,064 (14.2)	1,593 (20.7)	<0.001
Hemorrhagic stroke	275 (0.6)	75 (1.0)	0.002
Chronic kidney disease	1,111 (2.6)	473 (6.1)	<0.001
Liver cirrhosis	376 (0.9)	101 (1.3)	<0.001
Cancer	3,210 (7.5)	985 (12.8)	<0.001
COPD	2,278 (5.3)	769 (10.0)	<0.001

*Wilcoxon rank-sum test was performed for continuous variables. The chi-squared test was used for categorical variables. ICH, intracerebral hemorrhage; COPD, chronic obstructive pulmonary disease
